# Nasopharyngeal Branchial Cleft Cyst: A Rare Case Report and Literature Review

**DOI:** 10.7759/cureus.43432

**Published:** 2023-08-13

**Authors:** Suzan Alzaidi, Omar A Alsulami, Saad Alqarni, Alshema Alqurashi, Abdullah Ghafouri, Elham S Bin Abbas

**Affiliations:** 1 Otolaryngology - Head and Neck Surgery, King Fahad Armed Forces Hospital, Jeddah, SAU; 2 Otolaryngology - Head and Neck Surgery, King Abdulaziz University Faculty of Medicine, Jeddah, SAU; 3 Pathology and Medical Laboratory, King Fahad Armed Forces Hospital, Jeddah, SAU

**Keywords:** ct, rosenmuller, nasopharynx, adult male, branchial cleft cysts

## Abstract

Branchial cleft cysts are birth defects that happen when the first through fourth pharyngeal clefts do not close properly and most of these cysts develop from the second cleft. Second branchial cleft cysts are almost always in the neck, so it is rare for them to present in the nasopharynx. We report an extremely rare case of a branchial cleft cyst that is located in an unusual site in the nasopharynx in a 36-year-old male with no prior medical history. Computed tomography scan findings showed non-enhancing thickening of the right side mucosal-pharyngeal space, obliterating the fossa of Rosenmuller with no invasion or erosion. The patient was admitted for nasopharyngeal mass excision, and the mass was sent for histopathology. When a cystic lesion is noted in the lateral nasopharynx, branchial cleft cysts should be on the list of possible diagnoses. Surgery is primarily the treatment. The marsupialization approach is a simple way to treat nasopharyngeal branchial cleft cysts as it is safe and has limited complications

## Introduction

Branchial abnormalities are the result of aberrant branchial apparatus development during embryogenesis, which occurs between the second and sixth-seventh weeks of fetal life. Branchial remnants that are still present can result in the growth of cysts, sinuses, fistulas, or cartilage islands [1]. Branchial cleft cysts (BCC) are congenital epithelial cysts and account for almost one-third of congenital neck masses. They can appear at any age and are benign lesions. The majority, referred to as second BCC, are visible as a non-tender fluctuant mass along the sternocleidomastoid muscle's anterior border. They often appear after an upper respiratory infection as a neck mass that waxes and wanes in time with the disease [2]. Even though BCC are present from birth, many cases do not manifest until later in childhood or adolescence, and it is uncommon to see an initial clinical presentation in adults [3].

BCC are frequently asymptomatic; however, they have tendency to grow, become tender, or become inflamed during episodes of upper respiratory tract infections, leading to the development of an abscess or secondary infections. In such circumstances, the patient may exhibit purulent sinus discharge to the skin or throat. Due to cyst compression of the upper airway, dysphagia, dyspnea, and stridor are the most alarming symptoms [4]. BCC might be suspected based on a patient's medical history and clinical symptoms; confirmatory imaging techniques include computed tomography (CT) scan, magnetic resonance imaging (MRI), ultrasonography, and fine-needle aspiration. Surgical excision is typically the mainstay of management. The diagnosis of this embryological defect is made easier by the location, clinical picture, and radiological correlate, as well as a high degree of suspicion for the condition [5].

Congenital BCC of the nasopharynx is quite rare and typically develops from the lateral nasopharynx with inferior and medial extension. The cysts often have a single, unilateral location and exude mucus. By means of their histopathologic presentation, imaging features, and particular nasopharyngeal localisation, they can be distinguished from other nasopharyngeal cancers [6]. We report an extremely rare case of BCC that is located in an unusual site in the nasopharynx and present a review of the literature of the sites of origin, clinical presentation, and different diagnostic and therapeutic modalities.

## Case presentation

**Figure 1:**
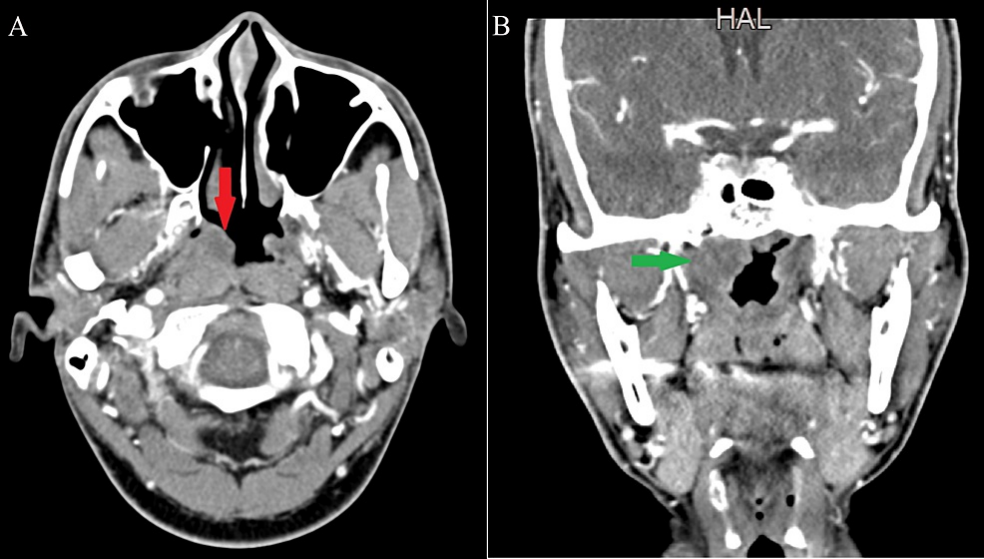
Computed tomography scan with contrast, A) axial cut showing right nasopharyngeal mass obliterating fossa of Rosenmuller (Red Arrow). B) coronal cut showing right nasopharyngeal mass obliterating fossa of Rosenmuller (Green Arrow)

A 36-year-old male patient suffering from allergic rhinitis visited the ENT clinic for regular check-up and medication refill. He was on PRN (administration as per need) treatment and had no other prior significant medical or surgical history. A nasopharyngeal scope was done for the patient, showing bilateral hypertrophy of the inferior turbinate and a nasopharyngeal mass on the right side of the nasopharynx obliterating the fossa of Rosenmuller. An enhanced CT scan of the neck with intravenous iodinated contrast was done for the patient. CT scan was preferred as the patient was claustrophobic and it was a faster procedure to follow. It showed non-enhancing thickening of the right side mucosal-pharyngeal space, obliterating the fossa of Rosenmuller with no adjacent soft tissue invasion and no erosion or vascular invasion (Figure 1).

**Figure 2:**
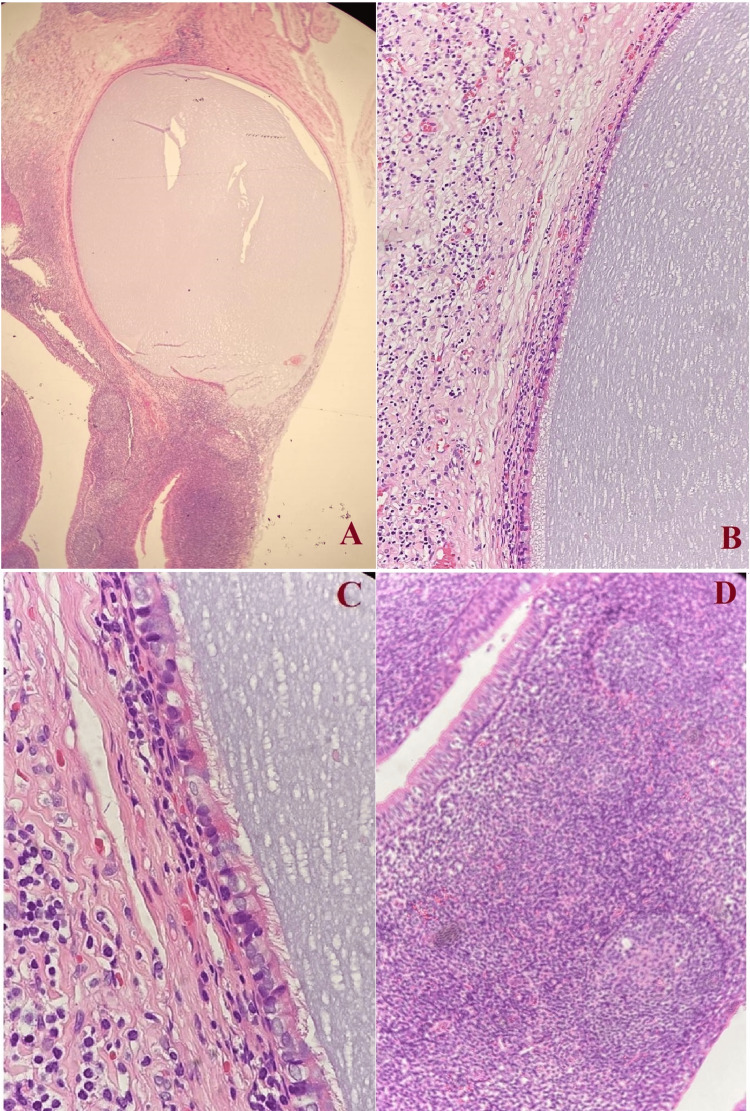
A) Photomicrograph slide showing cystic lesion lined by respiratory-type epithelium. Hematoxylin-eosin, magnification x40. B) Photomicrograph slide showing the cystic cavity lined by ciliated respiratory epithelium with underlying tissue showing lymph tissue. Hematoxylin-eosin, magnification x100. C) Photomicrograph slide showing the cystic lumen lined by pseudostratified columnar epithelium. Hematoxylin-eosin, magnification x200. D) Slide with hematoxylin-eosin stain showing underlying connective tissue with abundant lymphoid tissue and numerous reactive germinal centres. Hematoxylin-eosin, magnification x200

The assessment of the ear was normal with bilateral intact tympanic membranes. Additionally, the hearing sensitivity as per pure tone audiogram was also normal and type A bilateral tympanogram was observed. The patient was admitted for nasopharyngeal mass excision which was performed through cold steel straight forceps and direct nasopharyngeal scope zero degree under general anesthesia and removed with straight cup forceps and microdebrider. An incision into the cystic lesion under the view of rigid zero degree telescope through the nasal cavity was made. Multiple punch biopsies including full thickness of the cystic wall were taken for histopathological examination and the entire remaining lesion was cleaned out with the assistance of microdebrider. Grossly, the mass consisted of two fragments of grey-tan soft tissue. The first measured 2x0.5x0.5 cm and the second fragment measured 1x0.4x0.4 cm. Microscopic examination showed that the cystic cavity was lined by pseudostratified columnar epithelium underlying the connective tissue exhibiting lymphoid tissue and the cystic lumen was lined by ciliated pseudostratified epithelium (Figure 2).

A detailed literature review for reported nasopharyngeal BCC is reported in Table 1.

**Table 1:** Literature review of nasopharyngeal BCC in adults BCC: Branchial Cleft Cyst, CT scan: Computed Tomography scan, MRI: Magnetic Resonance Imaging.

Author	Age (years)	Gender	site	Clinical signs and symptoms	Imaging modality	Histopathology	Treatment
Shaheen -1961 [7]	18	male	bilateral	Nasal obstruction, nasal discharge	x-ray	Stratified squamous epithelium	Aspiration
	59	male	bilateral	----	----	Pseudostratified ciliated columnar epithelium	Excision
Singh KP and Pahor -1977 [8]	33	Male	midline	Snoring, mouth breathing, nasal discharge	CT scan	Squamous and transitional epithelium with salivary gland and lymphoid infiltration	Excision
Canty and Dogra-1978 [9]	57	Male	right	----	----	Pseudostratified ciliated columnar epithelium	Excision
Boysen et al-1979 [10]	45	female	right	----	----	Stratified squamous epithelium	Excision
Yoshimura et al -1986 [11]	58	male	right	Abnormal sensation in the nasopharynx	----	Stratified squamous epithelium	Excision
	48	female	right	Hearing impairment and ear fullness	----	Ciliated columnar epithelium	Excision
Takimoto et al-1989 [12]	12	male	right	----	----	columnar epithelium	Excision
Dilkes et al-1990 [13]	42	Male	right	----	----	Not reported	Excision
Frank A and Papay -1994 [14]	29	Male	Right	Hyponasal speech, dysphagia, and odynophagia.	CT scan	squamous epithelial lined cyst with lymphoid aggregates	Excision
Tamagawa et al-1995 [15]	57	male	left	----	----	Pseudostratified ciliated columnar epithelium with subepithelial lymphoid aggregation	Endoscopic excision
Verma A et al- 2000 [16]	35	female	right	Snoring mouth breathing, and a persistent right nasal discharge	CT scan	branchial cyst lined with stratified squamous epithelium.	Excision
Tsung Yen Tsai -2010 [17]	43	Female	Right	Odynophagia	MRI	Stratified squamous epithelium with lymphocyte infiltration in subepithelial region	Marsupialization
	83	Female	Left	Dysphagia	CT scan	----	Observation (due to poor cardiovascular state)
	29	Female	Right	Odynophagia	MRI	Stratified squamous epithelium with lymphocyte infiltration in subepithelial region	Marsupialization
Po-ShaoChen -2012 [6]	44	Male	Right	Intermittent right-sided stuffy nose	MRI	stratified squamous cell and pseudostratified columnar cell epithelium with underlying abundant lymphoid tissue	Marsupialization
Kim YW 2013 [18]	56	Male	Right	Right nasal obstruction accompanied by a sensation of fullness in the right ear	MRI	lymphoepithelial cyst consistent with a branchial cleft anomaly	Marsupialization
Erica Haught 2020- [19]	78	Female	Right	Right-sided aural fullness	MRI	----	marsupialization

## Discussion

BCC is divided into four major types: first, second, third and fourth, which are further divided into sub-types. First branchial cleft cysts occur when the cleft between the first and second branchial arches does not close completely leading to two different problems, referred to as type I and type II anomalies. Type I anomalies only involve the ectoderm, while type II anomalies involve both the ectoderm and the mesoderm. Type I lesions are extremely rare [20]. About 95% of all branchial abnormalities are second BCC. They can be along or near the front edge of the sternocleidomastoid muscle or anywhere along a second branchial fistula that goes from the skin of the side of the neck, between the external and internal carotid, and ends in the palatine tonsil. They are further divided into four subtypes. Type I cysts are located at the anterior edge of the sternomastoid muscle and lie immediately beneath the cervical fascia. The most prevalent type is type 2 which is below the enveloping fascia and extends to great/major vessels. Extension to the lateral wall of the pharynx occurs in type 3 cases and the cyst is located between the internal and external carotids. Usually, type 4 is a columnar-lined cyst that is medial to the carotids and close to the pharyngeal wall [5,21]. Third BCC are rarely observed but they have been identified as the second most common birth defect in the posterior cervical area, after thyroglossal cysts [22]. Fourth BCC are extremely uncommon and only seen every once in a while. Most of the time, they show up as recurrent deep neck infections, abscesses, or acute suppurative thyroiditis [23].

We reviewed 21 articles, including 28 cases, published from 1961-2021. It was observed that no specific age group was associated with a high risk of nasopharyngeal branchial cleft. The youngest age of presentation was two years old, published by Shidara et al. [24], and Kim [18]. The oldest age of presentation among our included studies was a case of an 83-year-old female, reported by Tsai [17]. The radiological modality used in the majority of the cases was CT scan alone although in some cases both CT scan and MRI were used especially among the pediatric age group. Nasopharyngeal BCC was more predominant in males than females, with a ratio of 4:3. Our case of nasopharyngeal BCC was on the right side of the nasopharynx, which is consistent with the studies reviewed. The ratio was 19:4:3:1 right, left, bilateral, midline. The most widely used treatment modality was surgical excision of the cyst. Aspiration was performed in one case reported by Shaheen [7]. Another patient who was 83 years old was observed due to poor cardiovascular status [17].

Sonography is the first-line imaging technique of choice for diagnosing these lesions because the cysts are superficial. It doesn't involve exposure to ionizing radiation and is quick, affordable, and non-invasive. On CT scan, these lesions normally seem well-circumscribed and uniformly hypodense with thin walls in the absence of complications; wall thickness may rise following an infection. A more thorough preoperative evaluation and a clearer representation of the cyst's deep extent are provided by MRI. In T1-weighted scans, the cysts' content ranges from being hypo- to isointense while in T2-weighted sequences, it is hyperintense [25]. The CT scan findings in our patient revealed non-enhancing soft tissue thickening of the right side mucosal-pharyngeal space, obliterating the fossa of Rosenmuller with no invasion or erosion. However, Ben Salem et al. described that when it comes to imaging for nasopharyngeal BCC, MRI with contrast is better than CT. Even though CT does a good job of showing how lesions relate to bone structures, MRI is better at showing how the lesion relates to the major blood vessels. Lesions of the nasopharynx can also be told apart by the way the signals look on an MRI. CT usually shows a mass that is not very dense [26].

Rare branchial cleft-derived lateral nasopharyngeal cysts are extremely uncommon, and the treatment is primarily surgical. No matter the approach used for treatment of nasopharyngeal cysts, the main objectives are to lessen the severity of the symptoms, exclude malignancy, and prevent bleeding and cyst recurrence. Nasopharyngeal BCC can be treated by aspiration, excision, or marsupialization. Successes with minimally invasive endoscopic/robotic techniques have been documented in several case studies. Nasopharyngeal cysts can be successfully treated with transnasal endoscopic marsupialization, which has great cosmetic and functional outcomes. An accurate excision of the lining, preservation of Eustachian tube function, and prevention of internal carotid damage at the lateral aspect of the cyst are all possible using a two-handed surgical method [19]. When compared to aspiration, endoscopic cyst marsupialization has the advantages of a lesser risk of internal carotid artery damage and a lower likelihood of cyst recurrence. Using an endoscopically guided diode laser for marsupialization, transnasal endoscopic marsupialization of a nasopharyngeal BCC has previously been described with no cyst recurrence, reported six months after surgery. When compared to transoral, transpalatal procedures, transnasal marsupialization has been shown to have advantages such as superior cosmetic appearance and less wound-related problems and pain [6,27].

Tsai and Su described the transoral cyst marsupialization for the three patients who were treated successfully with the surgery. The average time of follow-up was 21 months (range, 8 to 40 months). No clear complications or recurrences were seen after the surgery. Transoral marsupialization is a way to treat nasopharyngeal BCC that is easy, effective, and less invasive [17]. Similarly, Flis and Wein reported three cases of nasopharyngeal BCC that were treated surgically with an endoscopic transnasal marsupialization. During a follow-up period of one to four years, there was no recurrence. The surgery was done as an outpatient procedure and had no major complications [27]. Kim et al. stated that a simple, effective, and less invasive way to treat a nasopharyngeal BCC is powered instrument marsupialisation. Authors further reported two very rare cases of a nasopharyngeal BCC that caused symptoms and were treated with a powered instrument. Over two years of follow-up, there were no recurrence in any case [18]. Hence marsupialization approach seems to be most effective as per the literature, however, further research can be beneficial in studying its safety and efficacy and profile.

## Conclusions

Nasopharyngeal BCC is a rare lesion that can be observed as an incidental finding however it can also present with nasal obstruction symptoms. Histopathological findings confirmed the diagnosis of nasopharyngeal BCC in our case. Therefore, the location of the lesion and histopathological diagnosis is crucial for the diagnosis of nasopharyngeal BCC. CT scan is sufficient for assessment and diagnosis while MRI can be used for pediatric patients. Surgical excision is the most effective treatment for management and to rule out serious differential diagnoses.

## References

[1] Papadogeorgakis N, Petsinis V, Parara E, Papaspyrou K, Goutzanis L, Alexandridis C (2009). Branchial cleft cysts in adults. Diagnostic procedures and treatment in a series of 18 cases. Oral Maxillofac Surg.

[2] Barest GD, Mian AZ, Nadgir RN, Sakai O (Soto JA, Lucey BC). Traumatic and nontraumatic emergencies of the brain, head, and neck. Emergency Radiology.

[3] Nahata V (2016). Branchial cleft cyst. Indian J Dermatol.

[4] Possel L, François M, Van den Abbeele T, Narcy P (1997). [Mode of presentation of fistula of the first branchial cleft]. Arch Pediatr.

[5] Bagchi A, Hira P, Mittal K, Priyamvara A, Dey AK (2018). Branchial cleft cysts: a pictorial review. Pol J Radiol.

[6] Chen PS, Lin YC, Lin YS (2012). Nasopharyngeal branchial cleft cyst. J Chin Med Assoc.

[7] Shaheen OH (1961). Two cases of bilateral branchiogenic cysts of the nasopharynx. J Laryngol Otol.

[8] Singh KP, Pahor AL (1977). Congenital cyst of nasopharynx. J Laryngol Otol.

[9] Canty DP, Dogra TS (1978). Branchiogenic nasopharyngeal cyst. J Laryngol Otol.

[10] Boysen ME, de Besche A, Djupesland G, Thorud E (1979). Internal cysts and fistulae of branchial origin. J Laryngol Otol.

[11] Yoshimura H, Fujiyoshi T, Kurono Y, Kawauchi H, Mogi G (1986). Two cases of nasopharyngeal cyst. Pract Otol (Kyoto).

[12] Takimoto T, Akemoto Y, Umeda R (1989). Pharyngeal cyst arising from second branchial cleft. J Laryngol Otol.

[13] Dilkes MG, Chapman J, Stafford ND (1990). Per-oral excision of a branchial cyst. J Laryngol Otol.

[14] Papay FA, Kalucis C, Eliachar I, Tucker HM (1994). Nasopharyngeal presentation of second branchial cleft cyst. Otolaryngol Head Neck Surg.

[15] Tamagawa Y, Kitamura K, Miyata M (1995). Branchial cyst of the nasopharynx: resection via the endonasal approach. J Laryngol Otol.

[16] Verma A, Sohail MA, al-Khabori M, Moosa M (2000). Nasopharyngeal cyst of branchiogenic origin: report of a case and review of the literature. Ear Nose Throat J.

[17] Tsai TY, Su CY (2010). Surgical technique of transoral marsupialization for the treatment of nasopharyngeal branchial cysts. Ann Otol Rhinol Laryngol.

[18] Kim YW, Baek MJ, Jung KH, Park SK (2013). Two cases of nasopharyngeal branchial cleft cyst treated by powered instrument assisted marsupialisation. J Laryngol Otol.

[19] Haught E, Stevens L, Turner M (2022). Endoscopic marsupialization of a nasopharyngeal branchial cleft cyst using a drug-eluting stent. Ear Nose Throat J.

[20] Finn DG, Buchalter IH, Sarti E, Romo T, Chodosh P (1987). First branchial cleft cysts: clinical update. Laryngoscope.

[21] Proctor B (1955). Lateral vestigial cysts and fistulas of the neck. Laryngoscope.

[22] Jeyakumar A, Hengerer AS (2004). Various presentations of fourth branchial pouch anomalies. Ear Nose Throat J.

[23] Valentino M, Quiligotti C, Carone L (2013). Branchial cleft cyst. J Ultrasound.

[24] Shidara K, Uruma T, Yasuoka Y, Kamei T (1993). Two cases of nasopharyngeal branchial cyst. J Laryngol Otol.

[25] Vidhyadharan S, Krishnan S, King G, Morley A (2012). Transoral robotic surgery for removal of a second branchial arch cyst: a case report. J Robot Surg.

[26] Ben Salem D, Duvillard C, Assous D, Ballester M, Krausé D, Ricolfi F (2006). Imaging of nasopharyngeal cysts and bursae. Eur Radiol.

[27] Flis DW, Wein RO (2013). Nasopharyngeal branchial cysts-diagnosis and management: a case series. J Neurol Surg B Skull Base.

